# In-sensor cryptographic signature generation to link a physical process and an immutable digital entity

**DOI:** 10.1038/s41928-026-01593-5

**Published:** 2026-03-24

**Authors:** Fernando Cardes, Sebastian Bürgel, Xinyue Yuan, Qianchen Yu, Arianna Rubino, Jihyun Lee, Raziyeh Bounik, Vijay Viswam, Andreas Hierlemann, Felix Franke

**Affiliations:** 1 Department of Biosystems Science and Engineering, Eidgenössische Technische Hochschule Zürich, Basel, Switzerland; 2 Gnosis Ltd, World Trade Center, Gibraltar, Gibraltar; 3 HOPR Association, Zurich, Switzerland; 4 D-ITET Microelectronics Design Center, Eidgenössische Technische Hochschule Zürich, Zurich, Switzerland; 5 Institute of Molecular and Clinical Ophthalmology Basel, Basel, Switzerland; 6 Faculty of Science, University of Basel, Basel, Switzerland

**Keywords:** Electrical and electronic engineering, Information technology

## Abstract

Data forgery and manipulation can undermine trust in areas ranging from science to journalism. Artificial intelligence will probably exacerbate this problem, degrading our ability to distinguish between artificially created data and real recordings (such as those taken with a video camera). Here we report a recording device technology that establishes a cryptographic link between a physical process (the act of recording) and an immutable digital entity (such as an entry in a public blockchain). Our approach relies on a monolithic cryptographic sensor that integrates a sensor device with a hashing unit (that continuously hashes pieces of recorded data) and a cryptographic signature unit (that signs the hashes using a public key encryption scheme). The system then uploads the signed hashes to a trusted public repository. We provide a proof-of-concept implementation with a voltage sensor for extracellular recording of cardiac myocyte field potentials that is fabricated using 180-nm complementary metal–oxide–semiconductor technology.

## Abstract (web summary)

A monolithic cryptographic sensor, which integrates a sensor device with a hashing unit and a cryptographic signature unit, can be used to create a recording system that provides a cryptographic link between a physical process and an immutable digital entity.

## Main

The ability to prove the authenticity of digital data is crucial to establishing the reliability of evidence and maintaining public trust and is essential in areas of society such as scientific research, journalism, law enforcement and historical preservation^1^. However, the advent of deep fakes (artificially created data) has begun to undermine trust in digital recordings. Future developments in generative artificial intelligence (AI) will probably lead to a situation in which fake digital recordings of most sensors can be created artificially with modest efforts. For instance, sophisticated AI tools already make it relatively easy to create fake photos and videos that are almost indistinguishable from real ones^2,3^. The availability of AI-generated data that are virtually indistinguishable from real recordings will also become a problem for the training of novel AI systems. Once databases used to train AI systems are significantly contaminated with AI-generated data, models may collapse and training may become inefficient^4^.

**Fig. 1:**
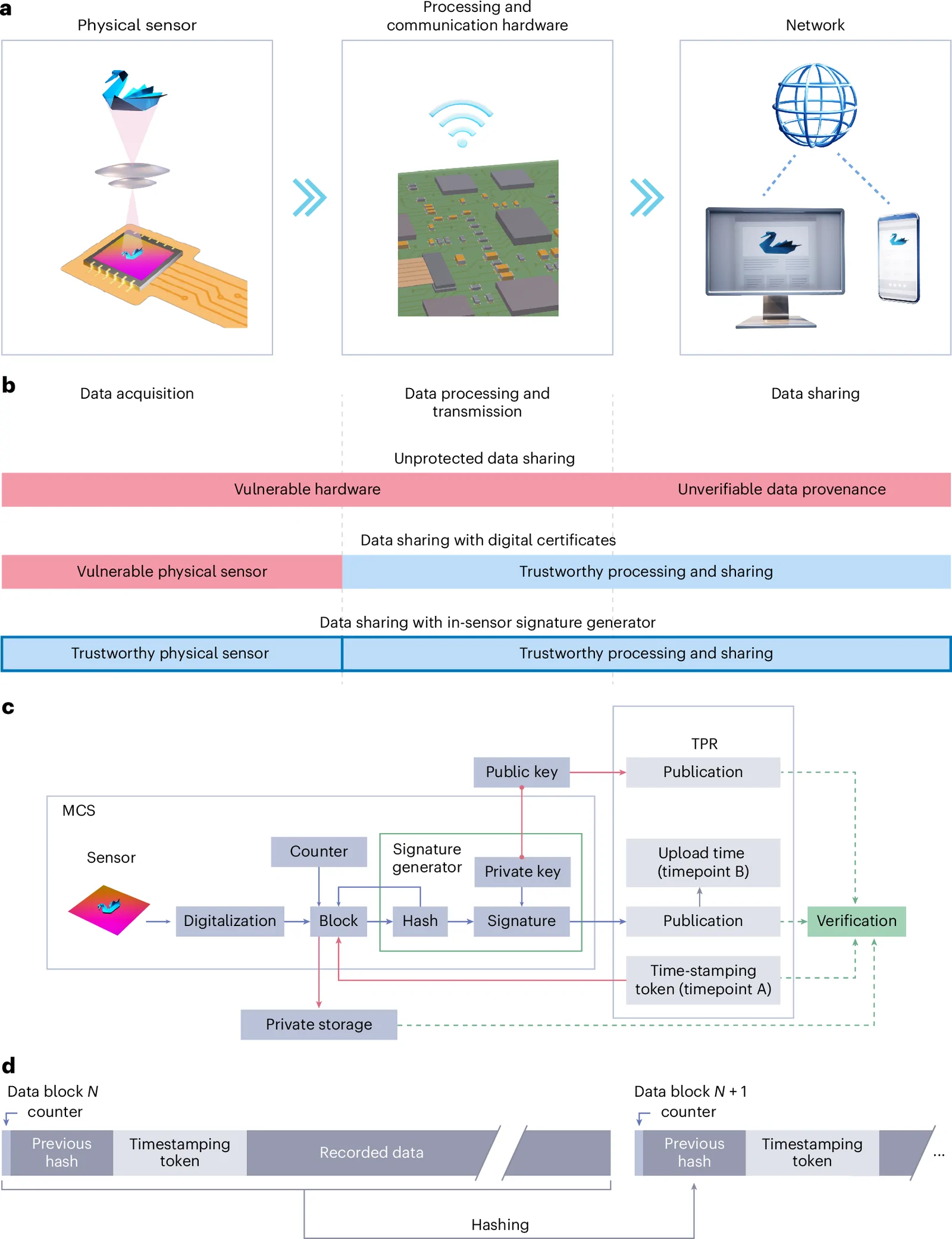
Overview of the system architecture. **a**, Simplified block diagram of a typical data acquisition system. A physical sensor, in this case a CMOS camera, generates digital data as a response to a physical stimulus (that is, light reaching the integrated light sensors). Digital data are sent to another IC and, after data processing, data are transferred to the network. **b**, Vulnerability assessment of three different data acquisition and sharing paradigms. In the most common scenario, data are acquired and shared without any reliable data provenance certificate, making data origin very difficult to trace. Digital certificates, created during data processing, make it possible to identify the hardware upon sharing a given recording. Integration of signature generators inside the sensor producing the original data drastically reduces the vulnerability of the system to physical attacks. **c**, Simplified schematics showing the monolithic cryptographic sensor (MCS) and a flow chart of the recording process. Some details are omitted, for example, the key generation process on the chip (the grey line connecting the keys indicates that this is a key pair) and the manufacturer, who retrieves the generated public key and publishes it on the trusted public repository (TPR). The arrow from ‘hash’ to ‘block’ indicates that each block contains the hash of the previous block. Colours indicate the sensor (light blue), the signature generator (solid green box), the verification process (dashed green lines) and the communication with the MCS (red lines). **d**, Schematic of the contents of a data block and its link to the next block.

To preserve trust in digital recordings, it is necessary to render the provenance of the data and their acquisition verifiable: that is, it must be possible for a third party to verify whether data, supposedly recorded with a real physical sensor, have instead been created or manipulated using AI. Cryptographic methods, such as those promoted by the Coalition for Content Provenance and Authenticity (C2PA), allow cameras and smartphones to digitally sign recordings, providing additional information on the provenance and editing history of digital content^5–7^. Furthermore, public blockchains can be used to improve the tracking and verification of online photographs^8^. However, to establish that data originated from a physical device, and were not generated by software, it is necessary to exclude the possibility that an attacker may feed fake data into the processing chain before the trust certificate is established (Fig. 1a,b). Injecting counterfeit data at the input of a cryptographic generator is relatively straightforward when the cryptographic generator is implemented in hardware that is separate from that of the sensor. In such cases, the digital data produced by the sensor pass through unprotected macroscopic connections, rendering it vulnerable to tampering. By contrast, integrating a cryptographic engine within the sensor itself, in the same integrated circuit (IC), would lower the risk of such attacks^9–12^.

In this Article, we report a recording system that provides an uncompromisable acquisition and digital representation of physical sensor data by relying on a monolithic cryptographic sensor (MCS), which cryptographically signs the data inside the sensor system that has been integrated on a single physical substrate. A prototype of the MCS is created with 180-nm complementary metal–oxide–semiconductor (CMOS) technology and monolithically integrates a sensor device with two additional units: a hashing unit that continuously hashes pieces of recorded data and a cryptographic signature unit that signs the hashes using a public key encryption scheme. The system then uploads the signed hashes to a trusted public repository (TPR), although this system could be combined with any other certificate system.

The monolithic implementation establishes a foundational layer of protection at the point of data capture. Existing approaches, such as trusted platform modules and other hardware-based security architectures, typically take effect only after data have been digitized, encoded and transferred within the recording device^7^. Consequently, these methods remain vulnerable during the transfer of digital signals between ICs, where unprotected physical connections may expose them to manipulation or injection of falsified data. By integrating cryptographic functions directly into the sensor’s silicon substrate, our monolithic design ensures data security from the source signal onwards. The central trust anchor of our system is the inaccessibility of the private key within the MCS, even for the owner and user of the system. As the main trust anchor relies only on the signature mechanism inside the physical substrate directly at the source of data creation, all other components of the system—such as the operating system, the connection to a public database and the user—do not need to be trusted.

## System architecture

Our cryptographic recording system comprises three elements: the recording device featuring the MCS, a TPR and a software-based verification tool (Fig. 1c). When the MCS takes a recording, the data are directly hashed and cryptographically signed using a public key encryption module within the MCS, and the resulting signature is subsequently uploaded to the TPR. The TPR provides a timestamp B, when the signature was uploaded. The verification tool can establish the authenticity of the recording, when it has access to the original recording, the public key belonging to the MCS and the location of the signature on the TPR.

The cryptographic sensor features two independent units for data acquisition and signature generation. The data acquisition unit can be designed according to the requirements of any digital sensor, adhering to specifications in terms of accuracy, cost, size and power consumption. It contains sensors, transducers and circuits required to measure and digitize the target quantity of interest. For example, in imaging applications, a cryptographic camera would include an MCS featuring light sensors (photodiodes). The MCS also would contain the analogue circuitry (the analogue front end), analogue-to-digital converters and a digital interface.

The signature generation unit consists of two different modules: the hashing module and the module implementing asymmetric encryption (Fig. 1c). This unit can generate a digital signature for pieces of recorded data, which we will refer to as a data block or simply ‘block’. A block can, for example, include an image, acquired by using the light sensor, or a certain number of frames of an acquired movie. The hashing module uses a one-way function to calculate—in real-time—a number from the recorded data block (the hash), which is much smaller than the data block. One-way functions have the property that their output can readily be computed from the input, but nothing about the input can be derived from the respective output. In particular, it is impossible to alter the data without completely altering the resulting hash. The hashing module compresses the data into a much smaller number, which represents the data, and which can be handled more efficiently by the signature module.

The MCS then cryptographically signs the hash using a standard public key signature method and one or several pre-installed private keys. The private keys are securely stored on chip and play a crucial role for various features of the architecture, including the privacy of the user and the ability of the recording system to deliver cryptographic proofs. We provide different choices for private key sets and their implications for the system in Supplementary Sections 5 and 6. Here, we assume a single key pair consisting of a private key, which was pre-installed on the chip by the manufacturer, and for which only a single copy in the MCS exists, and the corresponding public key, which was saved in a public repository by the manufacturer (manufacturer public key). The user of the cryptographic recording system has access to the recorded data, the public key and the signature but not to the private key. In fact, no one has access to the private key because only a single copy should exist in hardware in the MCS. This feature can, for example, be securely achieved by using a hardware-based key-generation mechanism on the MCS itself^13^, which is implemented by the manufacturer, and which permits reading out the public but not the private key. Without the private key, it is impossible to compute the signature off chip. Therefore, it is impossible to alter the data or the hash without breaking the signature. The user then uploads the signature to the TPR but can keep the recorded data private. The TPR registers the time of the signature upload (timepoint B) and stores it with the signature. If—at some future point in time—the user wants to prove the authenticity of the recorded data to a third party, they can provide the recorded data, the location of the public key and the location of the signature on the TPR to the third party. The system, as described so far, enables the third party to prove that the recording was created by the use of an MCS and that it was recorded before timepoint B (the time of the upload to the TPR).

Our system does more than certify that data existed before the upload time. By incorporating an external timestamping token into the on-chip hashing operation, each signed block can be proven to have been recorded after a specified timepoint A (token time) and before the upload timepoint B. To establish temporal structure within recordings, the MCS links consecutive blocks by hashing each block together with the previous block hash, and can include a monotonic counter so that resets and omissions become tamper evident (Fig. 1d). As a result, a verifier in possession of the data block, public key and repository entries can establish: the block originated from an MCS, it was recorded within the interval [A, B], the relative order of blocks and whether blocks are missing between two certified blocks (Supplementary Section 1).

These claims can be validated using a public verification procedure that does not rely on trusted software or infrastructure. Given a data block, the corresponding repository entry and the manufacturer public key, a verifier recomputes the hash from the data block and validates the published signature against this hash. If the signature is valid, the verifier can conclude that the data originated from a MCS linked to the public key and that it were recorded before the repository timestamp. When timestamping tokens, hash chaining and counters are included, the same procedure additionally establishes recording time bounds, block ordering and the absence of intermediate blocks. The verification algorithm is fully specified and can be independently implemented; detailed data formats, cryptographic operations and verification steps are provided in Supplementary Section 2.

## Prototype

**Fig. 2:**
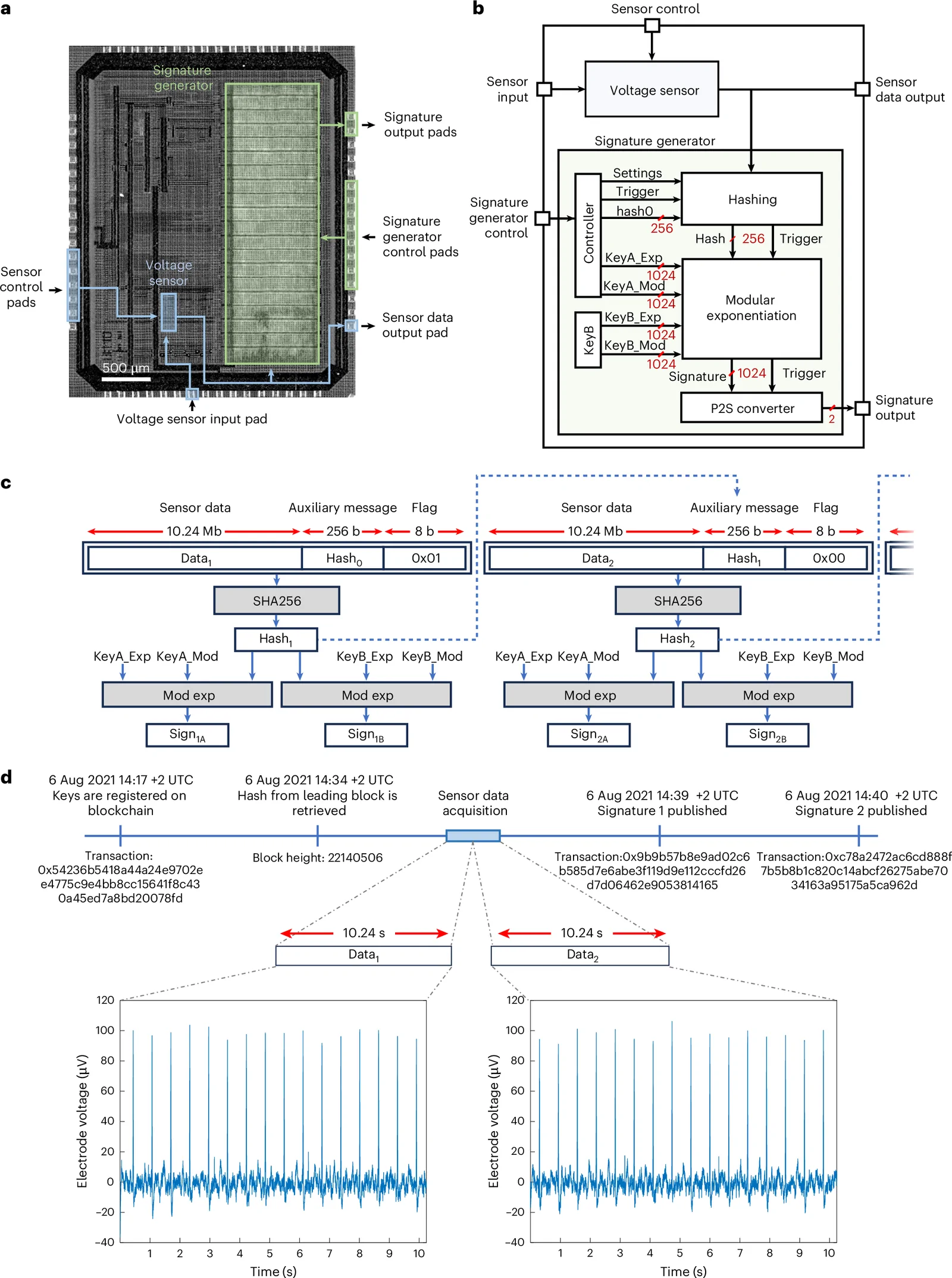
Details of the proof-of-concept implementation. **a**, Micrograph of the MCS. **b**, Simplified block diagram of the prototype. **c**, Data structure in the prototype. **d**, Timeline of the proof-of-concept bioelectrical signal recording. UTC, Coordinated Universal Time.

We designed and manufactured the MCS prototype in 180-nm standard CMOS technology. The micrograph of the 3 mm^2^ × 3.8 mm^2^ die is shown in Fig. 2a. Figure 2b shows a simplified block diagram of the MCS, which consists of a voltage sensor and a cryptographic signature generator. The architecture, circuits and performance of the voltage sensor are described in detail in previous work^14^. The sensor was optimized for measuring bioelectrical signals, such as neuronal and cardiac field potentials. The sensor produces a 1-Mbps bitstream on the basis of delta-sigma modulation. The sensed input voltage can be reconstructed from the output bitstream by means of digital postprocessing and band-pass filtering the bitstream to remove high-frequency quantization noise and low-frequency drifts.

The cryptographic signature generator consists of three modules: a hashing module, a modular exponentiation module and a parallel-to-serial converter (Fig. 2b). The hashing module calculates a 256-bit hash on the basis of the SHA256 algorithm. The hash is received by the modular exponentiation module, which computes two 1024-bit signatures using two different key pairs (KeyA and KeyB). The 1024-bit signatures are then packaged by the parallel-to-serial converter, which transmits the signatures off chip using a two-wire protocol. The cryptographic signature generator includes a controller module, which stores different settings, the initial hash and one of the keys (KeyA) in a volatile memory.

During the recording of a data block, the hashing module receives the data generated by the sensor and continuously updates the hash value until the block ends. We chose the SHA256 hash algorithm, a computationally efficient hash function, suited for hardware implementation and cryptographic applications. The module was designed to generate a hash for each data block (Fig. 2c), which consisted of 10.24 Mb of sensor data, acquired over 10.24 s at 1 Mbps; a 256-bit auxiliary message used to store either a timestamping token or the hash of the previous block; and an 8-bit flag indicating whether the device has been reset.

For the first hash of each measurement, the auxiliary message is written externally with the initial (timestamping) hash, and the 8-bit flag equals 0x01 to signify that it is the first block. For subsequent hashes, the auxiliary message is the hash of the previous block, and the 8-bit flag equals 0x00. We had to make some compromises in the implementation of the MCS and used the same 256-bit space for the token and hash. Instead of a persistent counter that would keep its value when the system was powered off, we used the 8-bit flag to differentiate between the first and subsequent blocks of a sequence of recorded blocks. In summary, the operations computed during hashing are

$${\mathrm{hash}}_{n}=\mathrm{SHA}256\left(\mathrm{concatenate}\left({\mathrm{data}}_{n},\,{\mathrm{hash}}_{n-1},\,{\mathrm{flag}}_{n}\right)\right),\,\mathrm{for}\,n{\mathbb{\in }}{{{\mathbb{N}}}},$$

where hash_0_ is the timestamping token retrieved from the TPR and

$${\mathrm{flag}}_{n}=\left\{\begin{array}{l}0{\rm{x}}01,\,\mathrm{if}\,n=1\\ 0{\rm{x}}00,\,\mathrm{if}\,n > 1.\end{array}\right.$$

For the signature, we implemented the RSA algorithm with 1024-bit key length, which is based on modular exponentiation. The modular exponentiation module receives the hash and a trigger signal from the hashing module every 10.24 s. Because the hash is only 256 bits in length, it needs to be padded before signing. For simplicity, we zero-padded the hashes (see discussion below on semantic security of the RSA algorithm and privacy concerns). For each data block, the hash is processed twice, performing modular exponentiation using two independent modulus–exponent pairs. Each modulus–exponent pair constitutes an RSA key. The first pair, namely KeyA, is stored in a volatile memory and can be programmed by the user dynamically. The second pair, KeyB, is hardwired in the CMOS prototype and cannot be read or modified externally. The exponentiation module first performs the following calculation:

$${\mathrm{Sign}}_{n{\rm{A}}}={{\mathrm{hash}}_{n}}^{\mathrm{KeyA\_Exp}}(\mathrm{mod}\,\mathrm{KeyA\_Mod}),\,\mathrm{for}\,n{\mathbb{\in }}{{{\mathbb{N}}}},$$

where KeyA_Exp and KeyA_Mod are the exponent and modulus of the private key part of KeyA. This calculation is performed in less than 500 ms. After that, the exponentiation module calculates the second signature as follows:

$${\mathrm{Sign}}_{n{\rm{B}}}={{\mathrm{hash}}_{n}}^{\mathrm{KeyB\_Exp}}(\mathrm{mod}\,\mathrm{KeyB\_Mod}),\,\mathrm{for}\,n{\mathbb{\in }}{{{\mathbb{N}}}}.$$

KeyA is not strictly necessary to implement the MCS but permits adding a second, user-supplied key, already at the hardware level. Such a user-supplied signature could also be added in software after the recording.

For our proof-of-concept implementation, we used the Sokol blockchain (an Ethereum-compatible blockchain for testing purposes) as the TPR. We implemented a software layer in Solidity that provided upload and verification functionality. First, we uploaded the public keys of both KeyA and KeyB. Immediately before starting the recording, the software layer retrieved the hash of the latest block of the TPR and stored it as the initial hash (hash_0_) in the auxiliary message space of the MCS (Fig. 2c).

After a data block was recorded by the MCS, the software layer published the corresponding signed hash to the TPR (Fig. 2d). It then retrieved the identifier of the corresponding block and stored it locally together with the recorded data block. The final recording consisted of a 3-tuple: the data block, the signed hash and the TPR pointer, which—in this case—was the transaction hash on the Sokol blockchain.

To experimentally validate the system under realistic sensing conditions, we used the prototype for bioelectrical recordings from in vitro cardiomyocyte cultures. The details regarding the assembly of the voltage sensor and the preparation of the biological samples can be found in ref. ^14^. The timeline of the recording is shown in Fig. 2d. Two consecutive blocks of 10.24 s of data were captured and signed. After that, the two signatures were published on the blockchain. On the basis of the publishing times of the different tokens, it can be claimed that the recording happened on 6 August 2021, between 14:34 and 14:40 Central European Summer Time (CEST) (Supplementary Section 3).

## Limitations of the prototype

The proof-of-concept implementation has several limitations that do not affect the conceptual validity of the approach but should be addressed in production systems. In the prototype, a timestamping token is embedded only in the first data block of each recording, which requires access to the full blockchain to verify the timing of individual segments; including tokens more frequently would enable independent verification of arbitrary blocks. For simplicity, the prototype zero-padded the hashes before generating the signatures, which is unsuitable for security-critical deployment; production systems should use modern, semantically secure signature schemes and key sizes consistent with current cryptographic recommendations.

Our prototype is not optimized for security or performance owing to resource limitations. The CMOS prototype was implemented in a 180-nm standard CMOS technology, which does not offer the best performance for digital circuits. As a result, the cryptographic signature generator occupied a comparably large area on our chip. Commercial sensors, implemented in smaller technology nodes, would feature a reduced area owing to the scaling of digital circuits (Supplementary Table 2 and Supplementary Sections 8 and 9). Moreover, the implementation of the hashing and modular exponentiation could be further optimized.

In terms of security, as the prototype did not include an on-chip key generator, we (as designers of the CMOS prototype) know the private keys and we could sign manipulated data (see Supplementary Section 4 for a detailed discussion on the limitations of the prototype). The prototype here is also not protected against reverse engineering, whereas a deployable MCS should incorporate multiple layers of defence against attempts to extract private keys. In addition, the prototype currently uses an 8-bit flag to indicate device resets. In production systems, this could be replaced with a tamper-resistant monotonic counter or other hardware-based time-tracking mechanisms, making it harder for an attacker to remove or reorder data blocks without detection.

Finally, the TPR used here is a test network, and the prototype lacks a verifiable binding between published public keys and specific hardware instances. Future implementations will require durable repositories and device-bound key generation to enable independent verification of genuine sensor-generated signatures. A detailed discussion is provided in Supplementary Section 4.

## Limitations, threats and privacy

The security of the system rests on three trust anchors: the inaccessibility of the on-chip private key, the integrity of the manufacturer public key and the immutability of the TPR. Under these assumptions, forging valid certificates for manipulated or synthetic data would require invasive hardware attacks, such as extracting the private key or injecting falsified signals within the monolithic substrate, which are technically challenging and costly. While denial-of-service or identity-level attacks may prevent successful publication or attribution, they do not enable the creation of false authenticity proofs. A systematic discussion of attack classes, including denial, identity theft and data-injection scenarios, together with mitigation strategies, is provided in Supplementary Section 5.

The system separates authenticity verification from data disclosure: only compact cryptographic signatures are published during recording, while raw sensor data are shared only when verification is desired. Nevertheless, continuous publication of signed hashes may reveal usage patterns, potentially affecting user privacy. This risk can be mitigated through repository-level anonymization, rotating identities, batching or delaying uploads and by avoiding inclusion of identifying metadata in signed data blocks. Importantly, signatures do not reveal information about the recorded content itself. Design trade-offs between verifiability, timing precision and user privacy, as well as strategies for anonymized provenance claims, are discussed in Supplementary Section 6.

Current cryptographic methods (including the RSA algorithm implemented in our prototype) rely on one-way functions, which are easy to compute in one way but computationally so expensive to invert that inversion can be considered impossible. However, advances in computer technology, especially the availability of quantum computers, may render such methods obsolete in the future. While such developments pose a serious problem for encrypted sensitive data, authenticity established by an MCS in the past will not be affected by these developments. The authenticity of the data is established at the time of the recording, not retrospectively. Thus, while a future attacker may be able to add further fake recordings, the authenticity of the data that were recorded before the security was broken cannot be retroactively compromised. This also holds in case the MCS is stolen. The thief would be able to make new recordings, which would not impact the authenticity of previous recordings. Furthermore, as the upload of the signature to the TPR is secured by a software-based user authentication scheme, the thief could not impersonate the original user.

## Conclusions

Our system provides a cryptographically secure way to prove that a specific set of digital data represents real physical recordings originating from a specific recording device. The user of such a device could cryptographically prove that: a given data set is a physical sensor/device recording and not a software-based data creation; a given data set originated from a single identifiable sensor/recording device; the recording was made before a specific timepoint; no recording was made between two subsequent recordings with the same device; and the recording was made after a specific timepoint (that of the previous recording).

The hardware-based cryptographic scheme can be considered strong, as an attacker would have to reverse-engineer the hardware to either extract the private key or insert fake data into the cryptographic module of the MCS. Extraction of the private key would already be difficult and expensive for standard hardware. However, it could be made even more difficult by using hardware protection schemes. The strength of the system relies on the fact that the sensor and the cryptographic module reside in the same physical substrate or microelectronic chip and that it is, therefore, nearly impossible for an attacker to insert a fake recording into the MCS and get it signed. The system could also be integrated into fully secure sensing applications using cloud computing (such as those using homomorphic encryption^15^ or zero-knowledge proofs^16^), where data integrity and provenance are ensured from the sensor level, embedded within the same physical substrate as the sensor.

The single-channel voltage sensor of this prototype generates 1 Mbps. However, in other applications, such as CMOS cameras, sensors can include millions of channels and generate massive amounts of data. Such applications may demand high-speed hashing circuitry, capable of processing real-time data rates exceeding Gbps. We investigated the feasibility of integrating our system into high-speed applications, such as real-time video streaming, and we conducted both a computational complexity analysis (Supplementary Section 7) and an assessment of the additional hardware demands (Supplementary Sections 8 and 9). The generation of the cryptographic signature is more resource-intensive than hashing and, therefore, poses a greater challenge.

A key design question concerns the smallest unit of information (data block) that requires certification. For example, if each video frame must be verifiable on its own, then every frame must be hashed and signed separately. Alternatively, if a single certificate suffices for a longer video segment, the system can process several seconds of data and generate one signature at the end of that segment. Our analysis revealed that a block rate of 60 Hz at gigabit-per-second data rates, as in live video streaming, appears feasible for efficient implementation in modern technology without entailing excessive power consumption or silicon area requirements.

Importantly, the system does not introduce latency in the data path because data can be transmitted off chip for publication immediately after acquisition, without waiting for hashing or signature generation (as the data itself do not require encryption). Signatures are available after a short delay, determined by the size of the data block, the smallest unit requiring certification.

Our prototype uses a basic voltage sensor, which digitizes an analogue voltage signal. However, the MCS architecture is designed to be highly adaptable and can support a wide range of sensor types. For example, a CMOS image sensor typically includes optics (such as lenses and filters), an array of photodiodes, amplifiers, multiplexers, analogue-to-digital converters and digital processing circuitry. In some implementations, all these components are integrated on a single substrate in a monolithic design. In other implementations, such as stacked configurations, the components are distributed across two or more dies. In these cases, the cryptographic signature should be generated on the die where analogue-to-digital conversion occurs because analogue signals are more difficult to authenticate and should be digitized before signing. An attacker might attempt to disconnect the dies and inject manipulated analogue signals at the interconnection pads. This could allow the attacker to obtain a valid signature, as the signature generator would be downstream of the injection point. However, such an attack would be technically demanding, requiring a cleanroom environment and the ability to inject thousands of analogue signals through microscopic connections. A less laborious method may involve displaying manipulated images on a screen in front of the camera. This manipulation approach also faces challenges, as the high resolution and sensitivity of modern sensors would reveal indications of tampering.

Even if individual sensors may be vulnerable to specific attacks, combining cryptographically signed data of multiple sensor types (such as temperature sensors, time-of-flight sensors or Global Positioning System modules) can improve tampering resistance. Cross-verification of data from different sources makes it much harder for an attacker, as they will have to manipulate all inputs consistently without leaving detectable traces.

## Supplementary information

Supplementary Information Supplementary Sections 1–4, Figs. 1 and 2 and Tables 1 and 2.

## Data Availability

Links to the data uploaded to the Sokol blockchain during the proof-of-concept (PoC) implementation can be found in Supplementary Section 3. Other data that support the findings of this study (including the electrophysiological recordings) are available from the corresponding authors upon reasonable request.
