# Supplementary material for: In-sensor cryptographic signature generation to link a physical process and an immutable digital entity
Source: Nat Electron. 2026 Mar 24;9(4):441–7. doi: 10.1038/s41928-026-01593-5 (PMC13649549; doi:10.1038/s41928-026-01593-5)
Supplement: Supplementary file 1 [file 41928_2026_1593_MOESM1_ESM.pdf]

nature electronics

Supplementary information

https://doi.org/10.1038/s41928-026-01593-5

In-sensor cryptographic signature generation to link a physical process and an immutable digital entity

In the format provided by the authors and unedited

Supplementary Section 1 – "Proof of temporal ordering and absence of recording"

As the user can delay uploading the signature, it is not possible to exclude that the user recorded the data at a much earlier time point. Thus, proving that a recording was performed after a specific time point A is a much more difficult problem than proving that it was recorded before time point B. Furthermore, proving the inexistence of a recording before time point A is a hard problem in general, since keeping a recording secret and pretending that it was created at a later time point is usually possible. We will now show how the system is able to prove that a recording was acquired after time point A. Indeed, the system is capable of providing proof of absence of recordings in between two time points, thus also proving the inexistence of recordings.

The MCS features the possibility for the user to attach a short piece of data to a data block during recording, which will be included in the hashing operation. The allocated space within the data block can be used to store a time-stamping token, i.e., a sequence of bits representing information, which verifiably existed only after time point A (Fig. 1C, D). The contribution of this token to the hash proves that the data block was recorded after time point A (token time). Examples of a time-stamping token could include the hashed website of a news agency, the latest prices of assets on the stock markets, the block hash of the latest block of a public blockchain, or any other source of regularly changing content that cannot be predicted. However, the token should be unequivocal, persistent, immutable, and should be easily accessible for verification. This token could be provided by the TPR or any trusted stable database. Because the signatures are now a function of the time-stamping token, which cannot have existed before time point A, it is possible to prove that the recording was performed between time points A and B (see Fig. 1C). If time point A is chosen to be right before the recording, and the signature upload is performed right after the recording, time point A (token time) and time point B (upload time) can, in principle, be arbitrarily close to each other, only limited by the recording time, the time to compute the signature, and the time necessary to download the token and upload the signature.

After hashing a data block, its hash is appended to the beginning of the subsequent block (Fig. 1D). As a result, the hash of each block depends on the hash of the preceding block, thereby linking the blocks and forming a cryptographic blockchain within the physical sensor substrate. This linking of the blocks also establishes the temporal order of an uninterrupted sequence of data blocks, such as consecutive frames of a video. A time-stamping token for time point A could be added only to the first block of a recording, since the cryptographic chain ensures that all subsequent blocks were recorded after the first block. However, this approach requires sharing the entire chain to verify that any particular segment was recorded after time point A. To overcome this limitation and enable independent verification, a time-stamping token can instead be added to every block (see Fig. 1D). Such an approach allows each block to be shared and verified separately, while maintaining proof that the block was recorded after a specific time point.

Furthermore, the MCS features a persistent monotonic counter, which is incremented with each recording block. The value of this counter is added to each data block before hashing and uploaded to the TPR together with the signature. The monotonic counter must be included in the data block before hashing so that resets become tamper-evident. It is thus possible to prove how many blocks were recorded in between two given blocks, and which of the blocks

was recorded first. This feature cannot be achieved with the time-stamping token alone, as the user could pre-acquire multiple tokens and then interchange the temporal order of the tokens and the recording blocks.

If a third party is in possession of a data block (including the counter, previous hash, time-stamping token, and recorded data), the public key, and the signature, they are able to prove

- that recording of the block was performed with an MCS
- that the block was recorded after time point A, indicated by the time-stamping token
- that the block was recorded before the upload of the signature (time point B)
- the temporal order of two or more blocks
- the inexistence of any recordings or the number of recorded blocks in between two given blocks.

Supplementary Section 2 – "Verification"

The TPR is a publicly accessible database into which the MCS can write. The entries in the TPR must be persistent, immutable, and time-stamped, and the user of the cryptographic recording system must be able to authenticate the provenance of the entries. For each recorded block, the MCS creates a new entry in the TPR including the signature. The TPR returns a pointer to the newly created entry, which the user of the MCS stores along with the recorded data block. The TPR data entry can be read by anyone in possession of the TPR pointer. Public blockchains typically meet all requirements of a TPR: they are publicly accessible, trustworthy, persistent, immutable, provide a time-stamp for each new entry, and can be used to generate an unequivocal time-stamping token (e.g., the block hash of the latest block of the blockchain).

The verification tool consists of a published verification algorithm together with a software implementation of that algorithm. The published algorithm contains a description of the data block structure (Fig. 1D), the data types and time formats, the meaning of the flags, the hashing algorithm used, and the cryptographic signature method. If a third party does not trust the provided software implementation, they can develop their own using the published algorithm. The tool enables any party to verify the correctness of an authenticity claim. To make such a claim, the user of an MCS must provide the location of the manufacturer public key published by the manufacturer, the TPR pointer, and the data block belonging to the TPR pointer.

The verification tool first checks with the manufacturer that the manufacturer public key is still valid. If a manufacturer key is compromised or decommissioned, the manufacturer can flag it (e.g., by entering it in the TPR with a revocation time point). The key status is revealed during the lookup. Importantly, TPR pointers created before the revocation remain valid, since authenticity is established at the time of recording and not retrospectively.

If the manufacturer key is valid, the verification tool re-computes the hash from the data block and decrypts the signature stored in the TPR entry using the manufacturer public key. The tool then checks whether the recomputed hash matches the decrypted signature. Finally, it outputs the comparison result together with the metadata.

This procedure only validates that the data was recorded with a device linked to the manufacturer's public key. It does not yet reveal the identity of the user, who generated the data, nor does it establish data provenance.

To provide proof of provenance, the user must also demonstrate ownership of the TPR entry. Thus, proof of provenance is linked to the TPR authentication system, whereas proof of authenticity is linked to the MCS and the manufacturer's public key.
If a device is compromised or decommissioned, the user can flag this with the manufacturer and obtain a replacement device. Continuity of data provenance is maintained via the user identification within the TPR.

To allow a third party to verify the authenticity of a recording, the user of the system provides the data block and TPR pointer to the third party, which can use the software layer to verify the existence of the signed hash on the TPR. The third party then recomputes the hash from the provided data block (H1). H1 needs to be compared to the signature in the TPR. In the RSA algorithm, a signature is equivalent to encryption, and verification is equivalent to decryption. Decrypting the signature using the public key, therefore, yields a second hash (H2). If H1 is equal to H2, the verification has been successful. In our PoC implementation, the time point of the recording can only be established for the first block of a recording, since only this block carries a time-stamping token. The user would, therefore, need to supply the uninterrupted sequence of blocks beginning with the first block, if they want to claim the time of a specific recording. The verifier can then check the validity of the time-stamping token in the first block to find time point A (token time). Time point B (upload time) is established by the time the signature was published on the TPR.

Fig 1C illustrates how the system could establish unfalsifiable evidence that a given photo was indeed recorded using a physically existing camera and neither created nor altered in software. The manufacturer fabricates the MCS, generates a single copy of the private key on the MCS, publishes the corresponding public key, and integrates the MCS into a camera. A user acquires the camera, the location of the corresponding public key, and a time-stamping token for time point A and takes a photo. The user publishes the signature of the photo in the TPR at time point B and subsequently shows the photo, e.g., on social media or as evidence in a court case. A third party challenges the authenticity of the photo or claims that it was taken after time point B. The user will provide the data block, the TPR pointer, and the manufacturer public key. Any interested party that is in possession of the original, unaltered photo, the signature, and the public key can prove - using the **verification tool -** that the photo was indeed taken between time points A and B using a camera of the respective manufacturer.

Supplementary Section 3 - "Verification in the proof-of-concept implementation"

In our proof-of-concept (PoC) implementation, the verification process required the following elements:

- A pointer (i.e., the transaction hash) to retrieve the public keys from the trusted public repository (TPR).
- A pointer to retrieve the initial hash from the TPR (i.e., the block hash of the latest block at the time of the recording).
- Pointers (i.e., the transaction hashes) to retrieve the signatures from the TPR.
- The data captured by the sensor.

- A detailed description of how the sensor data were processed to produce the hashes, including the method used to concatenate the initial hash (and any auxiliary data) and the specific hashing algorithm employed.

The Sokol blockchain was used as the TPR. An overview of all data uploaded to or read from this TPR is provided in Supplementary Table 1.

Data (Public KeyA_Mod)	0x8eb3614b4bed695f0bfd33a555279554035a1102dbf9a250191b9c5c2514cea367577d4634bcadf7e0d14ba2de3509a24ed3baab24e0d2f976b8c0e64fb8ff7c830453dc204ce6b1f4c82f3b8911ad005881ed16861091741ad92b45cd880158f0fa8b5dc10a6df226a7adab0cfe76831c5cad7733ec58c64ddc766b53ab2fb3
Data (Public KeyB_Mod)	0xb3a8a8c512faaa6e600fe9d0a78a14ebb82447c304889a034138332bf076f4fc0240a457d529db73be6e932e21176ff9ceaf58c03c362aeccfda2c61f6729ab9771957d5c6a9361c48a7cf6fc533e68f4341a3e198bb1740a0f5e31e3c7f3d34bd3910d121eebe3b4395e3b1aaac16deb084a765208bbf44c25499891f391e3b
Location	https://blockscout.com/poa/sokol/tx/0x54236b5418a44a24e9702ee4775c9e4bb8cc15641f8c430a45ed7a8bd20078fd
Description	Moduli of key pairs A and B
Data	0xa1d3f5d0751f7337bcfc3447a7d9961ebe6f170adb343d9dc419bc90e25e8319
Location	https://blockscout.com/poa/sokol/block/22140506/
Description	Initial hash
Data ($Sign_{1A}$)	0x585dfe1030e7eb8adc59f6513101de2715630b1031cc5ba4134def174c1c294c9b7d3b308c2ae3be546bcadba60fc2d0530d6f04f98ba8e23b23c1b4c1e0c32385a3bbe00928d2a5bd9a288aea1ca0cd0611833194db2c18e4b3550baa077b27ff7c4db494e174fda18965b86d76efa62c4f7de0ce8cc7939288a35d565655cb
Data ($Sign_{1B}$)	0x1204c60847f71a7029d8d34a2340464bec1276ae231a1d1db215684137aa5daa44c3887472df3dda21e09784c08505114e942520118835203f04ce4cdf3081486234f58f5ffa53f2b276c9f8d90988aa458bbea71626e0052157459fb5c525a1e5cbe223c4fc49dd0976fa14791fef4f32893c5748907a565c8e4258cc62684f
Location	https://blockscout.com/poa/sokol/tx/0x9b9b57b8e9ad02c6b585d7e6abe3f119d9e112cccfd26d7d06462e9053814165
Description	Signatures (A and B), corresponding to the first data set ($data_1$).
Data ($Sign_{2A}$)	0x045072df3358f433057fa0765e303e8b1e4c2077f1b8b564312928a5bf2e775c4f4d1cf8a957255f90146c567909c90247f44233142407a44378b7419590a8fc50033cfa5b31c9656073269b07d1c2028f86dcb6132c413ec7c2c8bf761d563b139283b4c7868237c9ef27fc573e05303c71718bd94acff341e5530e35b53003

Data ($Sign_{2B}$)	0x19ca1afde16b2452be9ae7a729927d9ee441ef31e566689f9e98ab8e3555 5ed549d2fd2cd6bf8d4aa3e2e6f3414ff3289ccf25fe481978281c21f309205f2 f7a27ffd674940ce3877152d6aed89881badf36dc1c88cf68394561171130e0 4271c423889f358e3618c2c5dbaf2dd16a052e8e495d5c68dbee8e84c1b84 6dbc59e
Location (transaction hash)	https://blockscout.com/poa/sokol/tx/0xc78a2472ac6cd888f7b5b8b1c820c14 abcf26275abe7034163a95175a5ca962d
Description	Signatures (A and B), corresponding to the second data set (data$_2$).

Supplementary Table 1. Summary of the data uploaded to the Sokol blockchain during the PoC implementation, including transaction hashes, block hash, timestamps, and associated signatures.

Supplementary Section 4 – “Limitations of the prototype”

A limitation of the prototype is that a time-stamping token is embedded only in the first data block of each recording. While this feature demonstrates the feasibility of linking data blocks to a specific time point, it also means that verifying the recording time of any given segment requires access to the entire chain of blocks from the beginning, which restricts independent verification of the timing of individual blocks or segments. More frequent use of time-stamping tokens — potentially in every block — would allow the verification of individual data segments without requiring access to the full recording.

Our prototype uses RSA-1024 with zero padding for simplicity of integration and proof-of-concept testing. This choice is not intended as a recommendation for deployment and is unsuitable for security-sensitive environments. For production systems, standard and modern digital signature schemes should be employed. For example, RSA-PSS (probabilistic signature scheme) introduces randomized padding, which mitigates the structural weaknesses inherent in naïve deterministic schemes, such as zero padding. Such a design provides strong resistance against adaptive chosen-message attacks and is standardized in PKCS#1 v2.2. Alternative signature schemes, such as Ed25519 or ECDSA-P256, also provide efficient, high-security elliptic-curve signatures, although the required silicon area and power consumption need to be considered. Key sizes should follow contemporary security recommendations (for example, at least 2048-bit RSA keys).

Finally, the trust infrastructure used in this prototype has important limitations. The TPR employed is a test net, and its long-term persistence and robustness cannot be guaranteed. Future production systems should, therefore, rely on more stable and durable infrastructure for publishing and retrieving verification information, such as the main Ethereum blockchain. More fundamentally, since we are user and designer of the prototype, we are in possession of the private key, and for a third party, there is no verifiable binding between the public keys published in the TPR and the private keys inside the cryptographic sensor. As a result, in this prototype, a verifier has no independent basis to determine whether a public key obtained from the TPR genuinely corresponds to a specific hardware instance. Addressing this limitation for a production system will require mechanisms that bind cryptographic keys directly to the device, enabling verifiers to identify genuine sensor-generated signatures.

Supplementary Section 5 – "Limitations and potential threats"

The cryptographic recording system that we described can be regarded as extremely secure although this does not necessarily hold for all components of our PoC implementation. As long as the three main trust anchors (the private key, the manufacturer, and the TPR) remain intact, we regard it as infeasible to tamper with previously uploaded signatures. However, several attacks could be carried out against the system, which we will group into three categories: denial, identity theft, and fake data injections.

Denial: The system as described can make use of unsecured public internet connections to upload the signed hashes. This way of operation would principally enable an attacker to interrupt the transmission or to modify the signature before it is written in the TPR. A missing or invalid signature would deny users of the MCS the ability to prove the authenticity of the recording. However, denial attacks do not enable the attacker to create valid entries in the TPR.

Identity theft: If an attacker attains access to the user credentials, used for the TPR, they could claim to be the originators of the recordings. However, such attacks could be easily averted by using secure software-based user identification schemes for uploading to the TPR. In our proof-of-concept implementation, such attacks could be rendered infeasible by protecting the private key of the blockchain with which the signature uploads were performed. Because our prototype MCS features a second, user-written private key to double-sign the hashes, this key could also be used to prove the provenance of the recording.

Fake data injection: The most severe attacks against the system could include fake data injections aimed at creating valid published signatures for artificially generated data. Assuming that the integrity of the TPR would stay intact, all these attacks would require the attacker to gain physical access to the MCS. With physical access, an attacker could also try to read out the private key from the MCS. While reverse-engineering a CMOS device is technologically feasible, such an attack would be extremely costly, and hardware-based protection schemes for data in such sensitive chips exist [1]. Alternatively, an attacker could try to inject fake data into the MCS between the sensor and the cryptographic module. Such an injection, again, would be very challenging to realize if the MCS was manufactured using modern CMOS technology, especially if the circuit and hardware of the MCS were designed with such attacks in mind.

The most likely attack against such a system would probably be of an entirely different nature and include using the MCS to perform a recording, but to tamper with the physical quantity, e.g., light or electric fields that the sensor records. An example could include pointing a camera at a computer screen on which an artificially created photo or video is shown. Such a recording of a fake image would create a valid signature in the TPR. The possibility to detect such an attack using data forensics on the raw data would depend on the nature of the sensor and the physical quantity that was recorded and would be independent of the cryptographic scheme, used to safeguard the recording or processing of the digital data. In the case of photos, simple attacks using consumer-grade displays would most likely be easy to detect.

In its simplest implementation, an MCS features one private key installed in the device during the fabrication of the CMOS sensor, and the corresponding public key is registered in the TPR

by the sensor manufacturer. During the process of generating and installing the key in the sensor, an attacker with access to the manufacturer's informatics system could be able to extract the private key. To prevent such an extraction, the MCS could include an on-chip key generator. In this way, the private key would never be available outside of the physical sensor device and the MCS would only send the public key off the chip on demand. One promising approach for on-chip key generation is the use of physical unclonable functions (PUFs) [2], [3]. PUFs exploit inherent manufacturing variations in CMOS devices to produce unique device-specific responses that can serve as cryptographic keys. However, it would still be necessary to trust that the manufacturer has not altered the design by manipulating the key generator or including a system that would make the private key easier to discover [1], [4].

Even if appropriate countermeasures against hardware trojans and similar attacks were in place, the integrity of the key pair could be compromised during the publication of the public key on the TPR. The person or system in charge of publishing the public key could replace the on-chip-generated public key with an externally generated public key for which the private key is known. To address this weakness, one may combine multiple keys. For example, an MCS could feature two private keys: one installed by the manufacturer (manufacturer key) and one generated on chip (device key). After generating the device key, the MCS could transmit off chip the device public key signed with the manufacturer private key. In this way, the entity that registered the public key of the device could not substitute the public key of the device without having the manufacturer's private key. Adding more keys, installed by different entities, would render the system more robust against this kind of attack.

Supplementary Section 6 – "Privacy"

The cryptographic security provided by our system comes at the cost of continuously uploading the signed hashes to a public repository. Such an activity could be monitored by an attacker, who could gain real-time insights into the usage pattern of the system, which could potentially compromise the privacy of the system user. Such a scenario could be particularly problematic for investigative activities under oppressive regimes, where the user would want to be able to publicly verify the authenticity of a recording while remaining anonymous. In the case of our proof of concept, it would be possible to link the origin of certain data or files to, e.g., a confiscated camera even at a much later time point.

Therefore, an important consideration is which information the cryptographic recording system may reveal about its users. For instance, if GPS coordinates were included to prove where the data were recorded, this could potentially expose the user's real-time location. Several design options can mitigate such risk and allow users to control how much information they want to reveal.

The system outputs two different data streams. The raw recording, which remains private with the users of the system until they decide to share the recording, and the continuously uploaded signed hashes. The raw recording only needs to be shared at the time when its authenticity should be verified and only with the party that should be able to perform the verification. Since this party could in principle be a trusted person or entity, we do not foresee any violation of user privacy.

It is important to note that the cryptographic security of signatures and the time stamping of uploads into the TPR are independent of the proof of provenance, which relies on the TPR's user authentication. Consequently, even if an MCS frequently uploads to the TPR, as long as

different user identities are employed for each upload, third parties cannot link the uploads to the user of the system. This linkage is only established at the time of verification.
Furthermore, it is not straightforward to locate an entry in the TPR without access to the TPR pointer. If the TPR is blockchain-based, a user could generate a new identity for each transaction, thereby obviating efforts to correlate multiple signatures to the same individual. Additional anonymization schemes could also be employed, for example, the restriction of database queries so that entries are revealed only when the correct TPR pointer is supplied. Importantly, it remains impossible for an attacker to infer information about the raw data, such as identity or location, from the uploaded signatures or hashes.
Obfuscation strategies to disguise usage patterns may include uploading dummy signatures, not associated with any recording, batch uploading multiple signatures, increasing block length, or randomly delaying uploads. These strategies, however, may come at the cost of increasing the time gap between the token time (time point A) and the upload time (time point B).
Care must be taken in the choice of the signature scheme. In our prototype, we zero-padded the hash before applying the RSA signature. This approach is not semantically secure and could allow an attacker to test manufacturer public keys against signatures. If such a test yields a zero-padded 256-bit value, the signature was likely created with the corresponding public key. Therefore, an attacker could identify the MCS from the TPR signature. To eliminate this vulnerability, production systems should employ stronger padding schemes that provide semantic security.
Another concern is that an attacker could attempt to link published data to a specific device. For example, with access to a user's camera, an attacker might try to determine which photos on social media were captured with that device. This risk would be amplified if each device carried a unique manufacturer key, and if the signature scheme lacked semantic security. Effective protection can be achieved by ensuring that individual public keys cannot be associated with specific signatures, as well as by assigning the same manufacturer key to multiple devices.
An alternative to verifying that a given signature is attributable to a specific device, zero-knowledge proofs [5] could potentially be employed to demonstrate that the data was produced by one of a set of authentic devices, without revealing which device it was. The integrity of such zero-knowledge proofs can be efficiently verified by the verifier software in the same manner as the signature verification described above.

Supplementary Section 7 - "Computational complexity analysis"

The two main operations of the system that determine computational complexity are the hashing operation and the cryptographic signature. The inputs to the system are the number of recording bits N, and the number of data blocks B into which we will split the N recording bits. That is, the number of bits per block (the block length) is $\sim\frac{N}{B}$ bits. We will consider each operation (hashing and signature) once for a fixed number of bits as a function of the number of blocks, and once for a fixed number of blocks as a function of the number of bits. We will assume that the number of blocks is meaningfully larger than 1 and that the block length is larger than 1.000 bits, and a multiple of 512.

Hashing operation:

The SHA-256 algorithm was designed to be computationally efficient. The input to the hashing operation is one recording block. SHA-256 divides each recording block into words of 512-bit length (within the SHA-256 context these are also referred to as blocks, but to avoid confusion, we will refer to them as words here). For each word, 64 rounds of operations are performed. Because we assume the block length to be a multiple of 512 bits, we will not consider scenarios in which zero-padding is necessary. If the number of bits, N, is fixed, the complexity of the SHA-256 algorithm is constant and independent of the number of blocks. That is, for practical purposes, it does not matter whether the algorithm is run many times on smaller blocks or fewer times on larger blocks. Since the SHA-256 mainly uses operations on 32-bit words and uses few operations per 512 bit word, it can be run online on the data during acquisition, and, for practical purposes, can be considered independent of the number of blocks, so that it is not a determining factor in the complexity of our system.

Cryptographic signature:
The signature is applied once for each private key to the hash of each data block. This means that the input to the signature is independent of the number of samples N_{fixed}, since only the 256-bit hash is signed. The complexity of the signature is, therefore, $O(B)$. However, the signature operation has a large constant cost, which dominates the overall complexity of our system. For example, the RSA signature of a k-bit private key requires a single modular exponentiation. Implementing modular exponentiation with a Montgomery ladder requires $2k(2k+1)$ additions of $\sim 2k$-bit numbers. That is, for a 1024 bit key, the system requires ~4.2 million additions of ~2048-bit numbers per private key per block.

Summary:
The system does not introduce additional latency to the raw data stream, as the data can be sent off the chip in parallel with the hashing and signature operations. The signature becomes available with a little delay with respect to the raw data depending on the length of a data block and the time necessary to compute the signature. The number of times the signature operation is applied is the determining factor in the overall computational complexity of the system. This number is linear in the number of private keys and linear in the number of data blocks, but independent of the bit rate or length of the recorded data. The constant complexity of the signature operation is quadratic in the bit length of the private key (when considering RSA using a Montgomery ladder).

Supplementary Section 8 - "Power and area estimation methodology"

The RTL code for the hashing and modular exponentiation blocks of our prototype implementation (see Section 6) was synthesized using the Synopsys Design Compiler Tool and standard cell libraries from the following technology nodes: 180 nm, 130 nm, 65 nm, and 22 nm. For each node, synthesis was performed under varying clock period constraints to explore trade-offs between performance and resource usage. Advanced technology nodes (such as 65 nm and 22 nm) offer a wider variety of standard cell libraries, with different threshold voltage and transistor length flavors. This diversity was exploited during synthesis to allow the Synopsys Design Compiler tool to choose the best set of libraries for different frequencies.

Dynamic power, leakage power, and area were estimated based on Synopsys Design Compiler synthesis reports. Dynamic power is the power consumed when the circuit is active, i.e., at any time that the voltage on a wire changes. Dynamic power includes charging and discharging of internal capacitances in the cell and short-circuit power (internal power), as well as logic transitions (switching power). Leakage power is the power dissipated when the circuit is inactive or static.

For each technology node, we estimated area and power requirements considering two clock frequencies. The first was the nominal frequency used in the prototype (1 MHz). The second was a higher frequency, specific to each node, representing an operating frequency that could be achieved without modifying the design. While even higher frequencies may be possible after a redesign, exploring such changes is beyond the scope of this work. We defined the high frequency value based on the estimates presented in Supplementary Figure 2, identifying the point at which further reductions in the clock period would lead to an exponential increase in both power consumption and required chip area.

Supplementary Section 9 - “Technology scaling and implementation overhead”

The area and power overhead of the prototype implementation of the cryptographic recording system are strongly influenced by the underlying CMOS technology and implementation choices. While optimization strategies (e.g., pipelining, parallelization, low-power design techniques) can significantly affect these metrics, such exploration is beyond the scope of this work.

Supplementary Table 2 summarizes the area, dynamic power, and leakage power synthesis estimations of the cryptographic recording system at the nominal frequency used in the prototype (1 MHz) and a higher operating frequency. Note that both area and dynamic power consumption decrease substantially with technology scaling. At 1 MHz the 22-nm implementation occupies approximately 30 times less area than its 180-nm counterpart, while consuming approximately two orders of magnitude less power. This trend is consistent for the technology nodes in between (130 nm and 65 nm).

Leakage power estimations are more dependent on implementation details. For example, to achieve 500 MHz clock rates using the 22-nm node, the Synopsys Design Compiler tool favors smaller-length and lower-threshold-voltage transistors leading to a leakage power of 35.1 μW and smaller area. For the same technology, at lower frequencies (1 MHz), the synthesis tool chooses a higher percentage of high-threshold-voltage and larger-length standard cells, resulting in a lower leakage of 0.5 μW and a larger area. The leakage power is typically orders of magnitude lower than the dynamic power. However, it should still be considered in low-power applications, where even small static losses can affect overall energy efficiency.

Supplementary Figure 1 shows the contribution of the hashing and cryptographic signature units in the total estimated dynamic and leakage power consumption and the overall required area. It can be observed that for all technologies, the signature unit is the main contributor both in terms of area and power requirements.

Supplementary Figure 2 shows that smaller nodes can reach shorter clock periods and, hence, support higher operating frequencies, enabling faster hashing and signature generation.

In summary, this study shows that our design can be operated across a wide range of clock frequencies, including values significantly higher than those used in the prototype. Such higher

frequencies are better supported by smaller technology nodes, which also offer substantial improvements in area and power efficiency. Even with a preliminary and unoptimized implementation in an older node, the system appears capable of performing real-time hashing and signing at rates above 100 Mbps, with a chip real estate of less than 2 mm² and a power consumption of less than 200 mW. While these figures may not yet meet the stringent requirements of some low-power systems, they are within the range for integration into devices, such as CMOS cameras. Overall, the results suggest that the current design is a promising starting point, and that further optimization can be done, guided by the specific constraints and requirements of the target application.

	Nominal frequency				High frequency			
Frequency (MHz)	1				125	250	333	500
Clock period (ns)	1000				8	4	3	2
Technology node (nm)	180	130	65	22	180	130	65	22
Operating voltage (V)	1.8	1.2	1.2	0.8	1.8	1.2	1.2	0.8
Area (mm^2)	1.75	0.93	0.27	0.06	1.9	0.97	0.28	0.05
Dynamic power (mW)	1.32	0.19	0.16	0.05	159	45.8	54.5	19.8
Leakage power (μW)	0.29	3.17	1.6	0.5	0.32	3.3	9.1	35.1

Supplementary Table 2. Estimated dynamic and leakage power consumption and area across four technology nodes.

Estimated area, dynamic power, and leakage power of the cryptographic recording system at the nominal frequency and a higher frequency for four CMOS technology nodes. Dynamic power, consisting of switching and internal power, leakage power and area were obtained from digital synthesis reports.

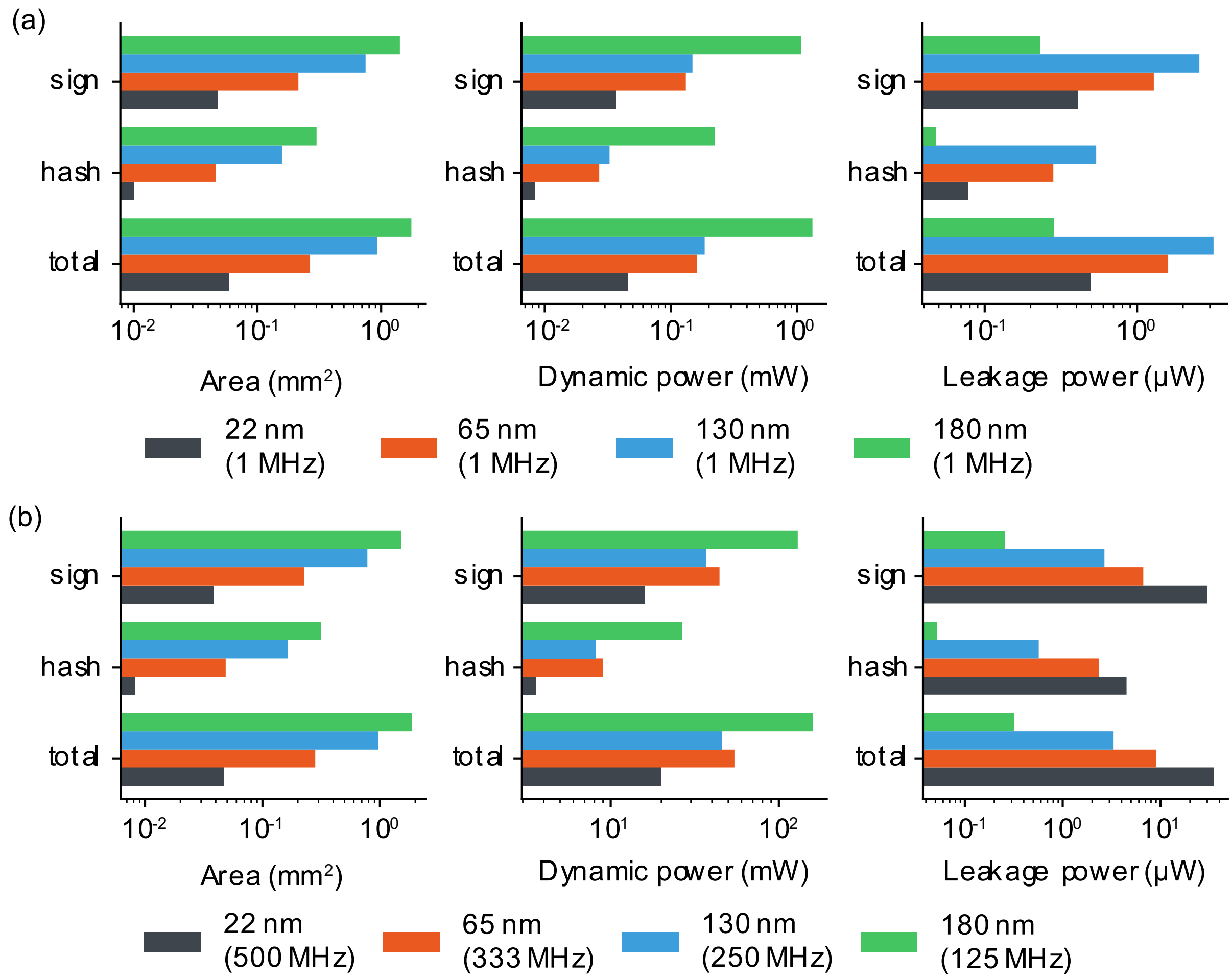

Supplementary Figure 1. Dynamic and leakage power and area breakdown of the main components of the cryptographic recording system across four technology nodes. (a) Breakdown for the operating frequency, 1 MHz. (b) Breakdown for the high operating frequency. Auxiliary circuits are not shown in the power and area breakdown due to their minimal contribution.

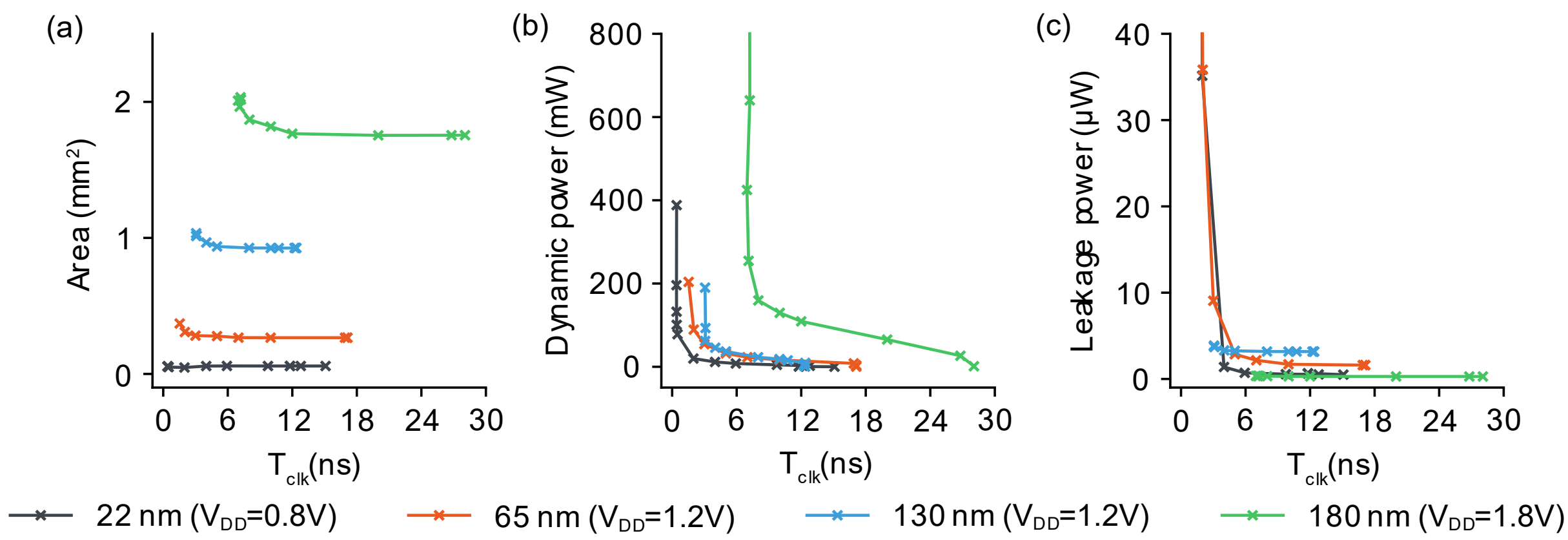

Supplementary Figure 2. Scaling of dynamic and leakage power consumption and area with clock period ($Tclk$) across four technology nodes.
Estimated area (a), dynamic power (b), and leakage power (c) of the cryptographic recording system as a function of the clock period for four CMOS technology nodes. Dynamic power, consisting of switching and internal power, leakage power and area were obtained from synthesis reports.

Supplementary References

[1] M. Rostami, F. Koushanfar, and R. Karri, “A Primer on Hardware Security: Models, Methods, and Metrics,” *Proc. IEEE*, vol. 102, no. 8, pp. 1283–1295, Aug. 2014, doi: 10.1109/JPROC.2014.2335155.
[2] R. Maes, “Physically Unclonable Function (PUF),” in *Encyclopedia of Cryptography, Security and Privacy*, S. Jajodia, P. Samarati, and M. Yung, Eds., Cham: Springer Nature Switzerland, 2025, pp. 1814–1817. doi: 10.1007/978-3-030-71522-9_1706.
[3] R. Maes, *Physically Unclonable Functions: Constructions, Properties and Applications*. Berlin, Heidelberg: Springer Berlin Heidelberg, 2013. doi: 10.1007/978-3-642-41395-7.
[4] R. S. Chakraborty, S. Narasimhan, and S. Bhunia, “Hardware Trojan: Threats and emerging solutions,” in *2009 IEEE International High Level Design Validation and Test Workshop*, IEEE, Nov. 2009, pp. 166–171. doi: 10.1109/HLDVT.2009.5340158.
[5] R. Lavin, X. Liu, H. Mohanty, L. Norman, G. Zaarour, and B. Krishnamachari, “A Survey on the Applications of Zero-Knowledge Proofs,” Aug. 01, 2024, *arXiv*: arXiv:2408.00243. doi: 10.48550/arXiv.2408.00243.
